# High expression of DEK predicts poor prognosis of gastric adenocarcinoma

**DOI:** 10.1186/1746-1596-9-67

**Published:** 2014-03-20

**Authors:** Junjie Piao, Yongjun Shang, Shuangping Liu, Yingshi Piao, Xuelian Cui, Yuzi Li, Zhenhua Lin

**Affiliations:** 1 Department of Pathology, Yanbian University Medical College, No. 977, Gongyuan-Rd, Yanji 133002, China; 2 Cancer Research Center, Yanbian University, Yanji 133002, China; 3 Department of Orthopedics, Affiliated Hospital of Chifeng University, Chifeng 024000, China; 4 Department of Internal Medicine, Yanbian University Hospital, No. 1327, Juzi-St, Yanji 133000, China

**Keywords:** Gastric adenocarcinoma, DEK, Immunohistochemistry, Survival analysis

## Abstract

**Background:**

DEK, as an oncoprotein, plays an important role in cancer development and progression. This study aimed to investigate the clinicopathological significance of DEK overexpression in patients with gastric cancer.

**Materials and methods:**

The expression of DEK protein was evaluated by immunohistochemical (IHC) staining of 172 gastric cancer samples with complete clinicopathological features, and the correlation between DEK expression and clinicopathological features was examined. Survival rates were also calculated using the Kaplan-Meier method in gastric cancer patients with complete survival data.

**Results:**

DEK protein showed a strictly nuclear staining pattern in gastric cancers with IHC and immunofluorescence. The strongly positive rate of DEK protein was 60.5% (104/172) in gastric cancers, which was significantly higher than that in either gastric dysplasia (19.4%, 7/36) or adjacent normal mucosa (0%, 0/27). DEK expression in gastric cancer correlated to tumor size, differentiation, clinical stage, disease-free survival, and overall survival rates. Further analysis showed that patients with early-stage gastric cancer and high DEK expression had shorter disease-free survival and overall survival duration than those with low DEK expression.

**Conclusion:**

High level of DEK protein expression predicts the poor prognosis of patients with gastric cancer. DEK expression might be potentially used as an independent effective biomarker for prognostic evaluation of gastric cancers.

**Virtual slides:**

The virtual slide(s) for this article can be found here: http://www.diagnosticpathology.diagnomx.eu/vs/5050145571193097

## Introduction

Gastric cancer is one of the most common cancers worldwide, and it is the second most common cause of cancer death [1]. In recent years, development of molecular drugs and traditional chinese medicine targeting oncogenic pathways has led to improvement of treatment outcome in gastric cancer [2]. In addition, the biomarkers for prognostic evaluation of patients with gastric cancers were also investigated widely. For example, Gao *et al.* reported that the expression of E-cadherin and claudin was correlated with lymphatic metastasis [3]. Geng *et al.* showed that the expressions of Pgp, GST-π and Topo II were related with gastric cancer chemosensitivity [4]. Furthermore, Canzonieri *et al.* also demonstrated that endocrine differentiation, maturely exocrine and endocrine gastric phenotypes are associated with poor prognosis [5]. However, the prognosis of gastric cancer has not significantly improved. Therefore, the discovery of novel biomarkers of gastric cancer is required for early diagnosis and to help provide novel therapeutic targets.

Human DEK was initially demonstrated to be the target of a recurrent t(6;9) translocation that generates fusion with CAN in a subset of acute myeloid leukemia (AML) patients [6]. It was identified as an oncoprotein and has a molecular weight of 42Kda. DEK plays an important role in cell processes and participates in a variety of cellular metabolic functions, such as global heterochromatin integrity [7], transcriptional control [8], mRNA splicing [9], DNA replication [10], DNA damage repair and susceptibility [11].

The roles of architecture proteins of chromatin in a variety of cellular mechanisms are dysregulated in tumor cells, such as altered expression levels and altered affinity to DNA, and result in occupancy of promoters and enhancers of other genes [12]. For example, Sanchez-Carbayo *et al.* reported that DEK was differentially expressed between the early-stage and invasive clusters of bladder cancer using cDNA microarrays [13]. Casas *et al.* detected DEK expression levels in 41 adult patients with AML using quantitative real-time PCR, and observed that DEK was overexpressed in 98% of cases [14]. We also previously found that DEK was significantly overexpressed in colorectal cancers and that the expression correlated to poor prognostic factors with colorectal cancers [15]. These results suggest that altered expression of DEK is associated with several human malignancies. However, the relationships between DEK expression and gastric cancer are not clear.

In the present study, we found that the expression of DEK was upregulated in gastric cancer tissues, and DEK might be an independent biomarker for the prediction of gastric cancer prognosis, suggesting that DEK plays an important role in the development and progression of gastric cancer.

## Materials and methods

### Ethics statement

This study complied with the principles of the Declaration of Helsinki and was approved by the human ethics and research ethics committees of Yanbian University Medical College in China. The patients were informed that their resected specimens were stored by our hospital and potentially used for scientific research, and that their privacy would be maintained. Follow-up survival data were collected retrospectively through medical-record analyses.

### Clinical specimens

Routinely diagnosed primary gastric cancer tissues (172 cases) with clinical features were collected from patients who underwent surgery between February 2002 and May 2004 by Shanghai Outdo Biotech Co. Ltd. (Outdo Biotech) and Tumor Tissue Bank of Yanbian University Medical College. Of the 172 cases, 67 were well differentiated, 82 were moderately differentiated, and 23 were poorly differentiated cancers. A pathological stage for each tumor was assigned using the Union for International Cancer Control (7th edition) criteria and World Health Organization classifications (Pathology and Genetics Tumors of the Digestive System) [16]. Total samples comprised 92 cases with stage 0–II and 80 cases with stage III–IV. Before surgery, no patients had received chemotherapy or had distant metastases. The follow-up time of the primary gastric cancer cohort was in the range of 5–8 years. By December 2012, 98 patients had died and 64 patients remained alive.

### Immunofluorescence (IF) staining for DEK in MKN-1 gastric cancer cell line

MKN-1 gastric cancer cells grown on coverslips was fixed with 4% paraformaldehyde in PBS for 10 min at room temperature and permeabilized with 0.5% TritonX-100 for 10 min. Then washed again with PBS and blocking was performed with 3% Albumin Bovine V (A8020, Solarbio, Beijing, China) for one hour at the room temperature (RT). Primary antibodies against DEK (1:50; BD Biosciences, USA) and β-Tubulin (1:50; Santa Cruz Biotechnology, USA) were incubated with cells at 4°C overnight. After more washes, cells were incubated with Alexa Fluor® 488 Goat Anti-Rabbit IgG (H + C) (A11008, 1:1000, Invitrogen, USA) and Alexa Fluor® 568 Goat Anti-Mouse IgG (H + L) (A11004, 1:1000, Invitrogen, USA) for 1 h. Subsequently, cells were washing again with PBS and counterstained with 2-(4-Amidinophenyl)-6-indolecarbamidine dihydrochloride (C1006, Beyotime, China). Coverslips were mounted with Antifade Mounting Medium (P0126, Beyotime, China) [17]. Finally, the immunofluorescence signals were visualized and recorded by Leica SP5II confocal microscope.

### Immunohistochemistry (IHC) for DEK in paraffin-embedded tissues

As described previously [15], a Dako LSAB kit (Dako A/S, Glostrup, Denmark) was used to perform immunohistochemical analysis. Four-micrometer-thick tissue sections were deparaffinized, rehydrated, and incubated with 3% H_2_O_2_ in methanol. Subsequently, the antigen was retrieved, followed by incubation with 1% bovine serum albumin. Slides were then incubated with a DEK antibody (1:50; BD Biosciences Pharmingen, San Jose, USA) at 4°C overnight. Normal goat serum was used as the negative control. After incubation with a secondary antibody at room temperature for 30 min, slides were incubated with streptavidin-peroxidase complex. The peroxidase reaction was developed with 3,3’-diaminobenzidine and counterstained with Mayer’s hematoxylin. Rabbit IgG isotope was used as a negative control and positive tissue sections were processed omitting the primary antibody as a further negative control.

### Evaluation of IHC staining

All slides were evaluated independently by two pathologists without prior knowledge of clinical outcomes. We first observed staining in the whole gastric lesion, and then quantified 50 representative fields. Only the nuclear staining pattern was considered as positive. The immunostaining was scored as ‘−’ (negative, no or less than 5% positive cells), ‘+’ (5–25% positive cells), ‘++’ (26–50% positive cells) and ‘+++’ (more than 50% positive cells). The strongly positive descriptor (DEK overexpression) was assigned to ‘++’ and ‘+++’ scored cells. For survival analysis, DEK expression level was denoted as high expression (‘++’ and ‘+++’) and low expression (‘−’ and ‘+’) [15].

### Statistical analyses

Statistical analyses were conducted using SPSS 17.0. Association between DEK expression and clinicopathological features were evaluated by Chi-square test and Fisher’s exact tests. The Kaplan–Meier method was used for analysis of survival curves, and statistical significance was assessed using the log-rank test. Multivariate survival analysis was performed on all significant characteristics measured by univariate survival analysis (gender, age, tumor size, differentiation, lymph node metastasis, serosal invasion, tumor stage, and DEK expression) through the Cox proportional hazard regression model. A *P* value *<*0.05 was considered statistically significant.

## Results

### DEK protein was overexpressed in gastric cancer

**Figure 1:**
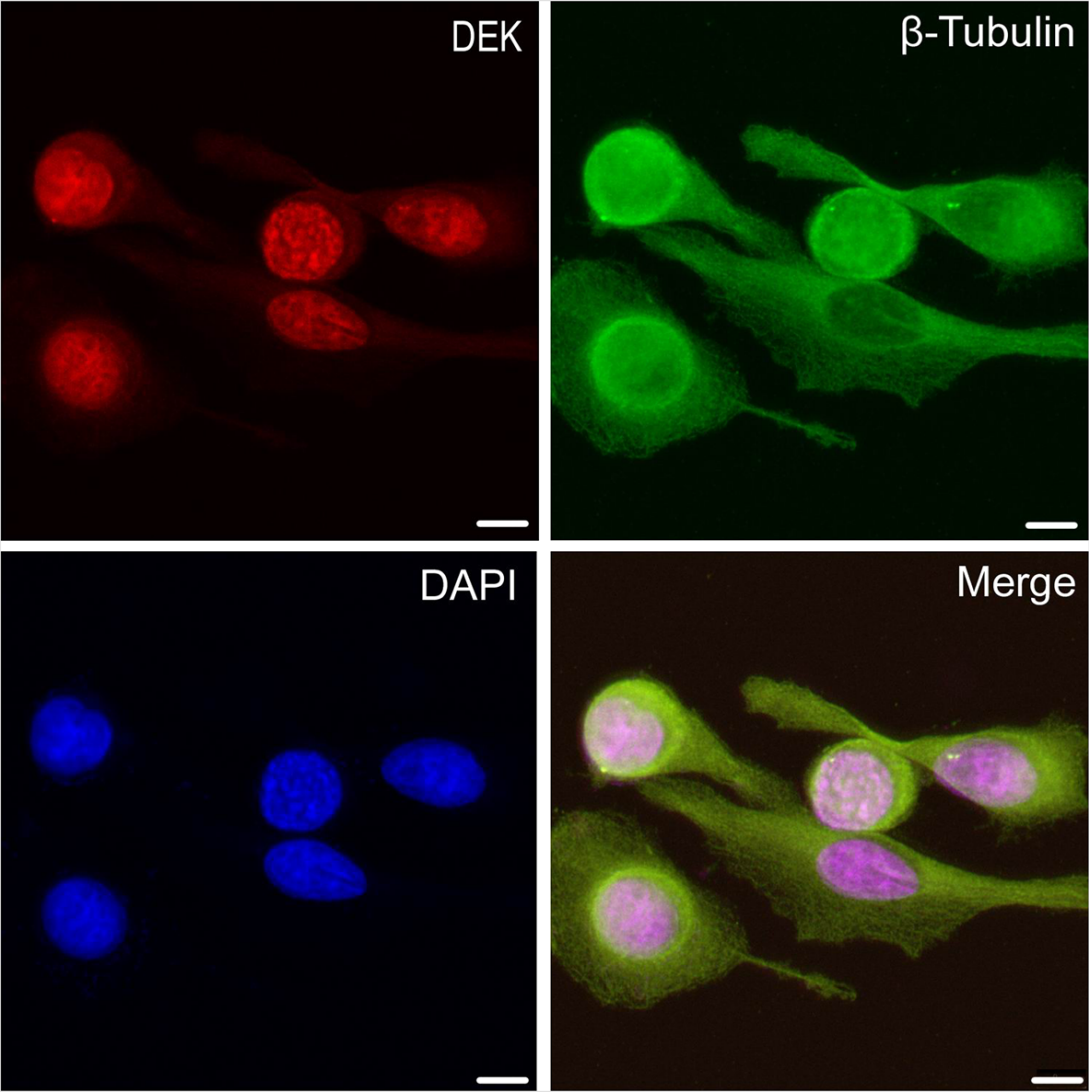
**Immunofluorescence staining for DEK and β-Tubulin proteins in MKN-1 gastric cancer cells.** DEK protein is strictly located at the nucleus of MKN-1 gastric cancer cells. (Red for DEK, Green for β-Tubulin, and Blue for DAPI).

**Figure 2:**
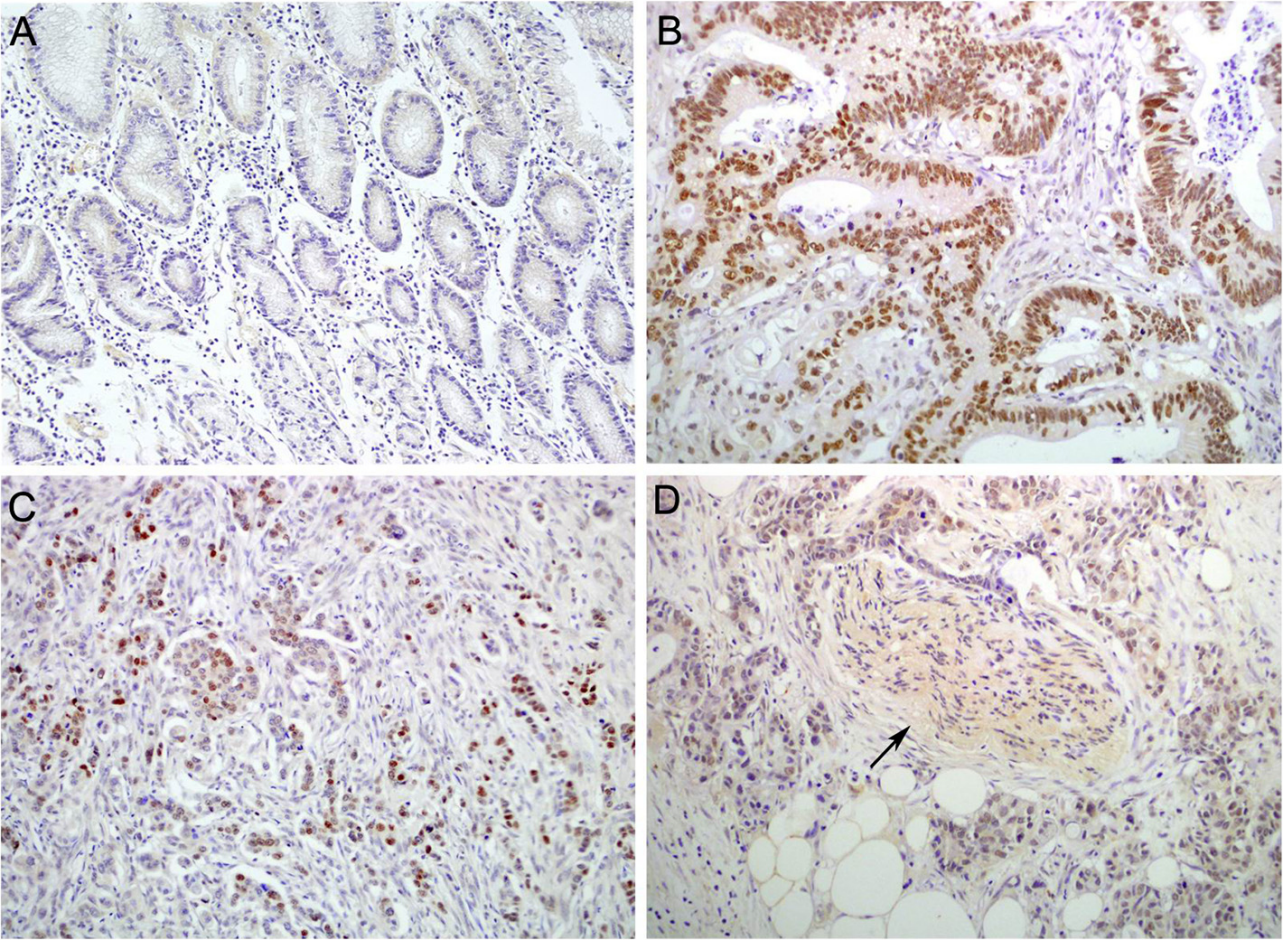
**Immunohistochemical staining of DEK protein in gastric cancer and normal mucosa. A**: DEK is negative in normal gastric mucosa. **B**: DEK protein is strongly positive in the nucleus of gastric cancer cells with lymph node metastasis. **C**: DEK protein showed scattered staining pattern in gastric adenocarcinoma without metastasis. **D**: DEK is positive in invasive cancer cells around the nerve (*arrow*) in the serosal layer (Original magnification, ×200).

DEK protein showed a strictly nuclear staining pattern in gastric cancers with IF (Figure 1) and IHC (Figure 2). The positive rate of DEK protein expression was 70.3% (121/172) in gastric cancer tissues, which was significantly higher than that in either gastric dysplasia (41.7%, 15/36) or normal adjacent mucosa (18.5%, 5/27). Similarly, the strongly positive rate of DEK protein (60.5%, 104/172) was also significantly higher than either gastric dysplasia (19.4%, 7/36) or adjacent normal gastric mucosa (0%, 0/27) (*P* < 0.01, respectively) (Table 1).

**Table 1:** DEK protein expression in gastric cancers

**Diagnosis**	**No. of cases**	**DEK expression**	**Positive rate (%)**	**Strongly positive rate (%)**
		**- + ++ +++**		
Gastric cancers	172	51 17 67 37	70.3%**	60.5%**
Dysplasia	36	21 8 7 0	41.7%**	19.4%**
Normal	27	22 5 0 0	18.5%	0

**P < 0.01, compared with normal mucosa and dysplasia of gastric. Dysplasia: gastric dysplasia; Cancer: gastric cancer; Positive rate: percentage of positive cases with ‘+’, ‘++’, and ‘+++’ staining score; Strongly positive rate: percentage of positive cases with ‘++’ and ‘+++’ staining score.

### Correlation between clinicopathological features and DEK overexpression

We analyzed the relationship between DEK overexpression and clinicopathological features of gastric cancers. The strongly positive rate of DEK expression was significantly higher in gastric cancers with ≥5 cm tumor size (55/73, 75.3%) than in cases with <5 cm tumor size (49/99 49.5%) (*P* < 0.05). Similarly, DEK expression was significantly higher in poorly differentiated gastric cancers (32/44, 72.7%) than in moderately (26/47, 55.3%) or well differentiated gastric cancers (46/81, 56.8%) (*P* < 0.05).

For TNM clinical staging, we found a strongly positive rate of DEK expression of 71.3% (57/80) in advanced-stage (III–IV) gastric cancers, and only 51.1% (47/92) in early-stage (0–II) cases (*P* < 0.01). Nevertheless, DEK overexpression in gastric cancers was not related to age, gender, Lauren types and serosal invasion (Table 2).

**Table 2:** Relationship between DEK protein overexpression and clinicopathological features of gastric cancer

**Clinical features**	**No. of cases**	**Strongly positive cases (%)**	***χ***^***2***^	***P *** **value**
**Age**			0.915	0.340
<56	96	55 (57.3%)		
≥56	76	49 (64.5%)		
**Gender**			0.011	0.918
Male	102	62 (60.8%)		
Female	70	42 (60.0%)		
**Tumor size**			10.501	0.001**
≥5 cm	73	55 (75.3%)		
<5 cm	99	49 (49.5%)		
**Serosal invasion**			0.283	0.596
Yes	81	46 (56.8%)		
No	91	48 (52.7%)		
**Lauren types**			5.581	0.118
Intestinal type	89	47 (52.8%)		
Diffuse type	72	51 (70.8%)		
Mixed type	11	6 (54.5%)		
**Differentiation**			19.732	0.000**
Well	67	27 (40.3%)		
Moderately	82	58 (70.7%)		
Poorly	23	19 (82.6%)		
**LN Metastasis**			1.417	0.235
Negative	89	50 (56.2%)		
Positive	83	54 (65.1%)		
**Clinical stage**			7.277	0.007**
0-II	92	47 (51.1%)		
III-IV	80	57 (71.3%)		

**P < 0.01.

### Correlation between DEK overexpression and survival of patients with gastric cancer

**Figure 3:**
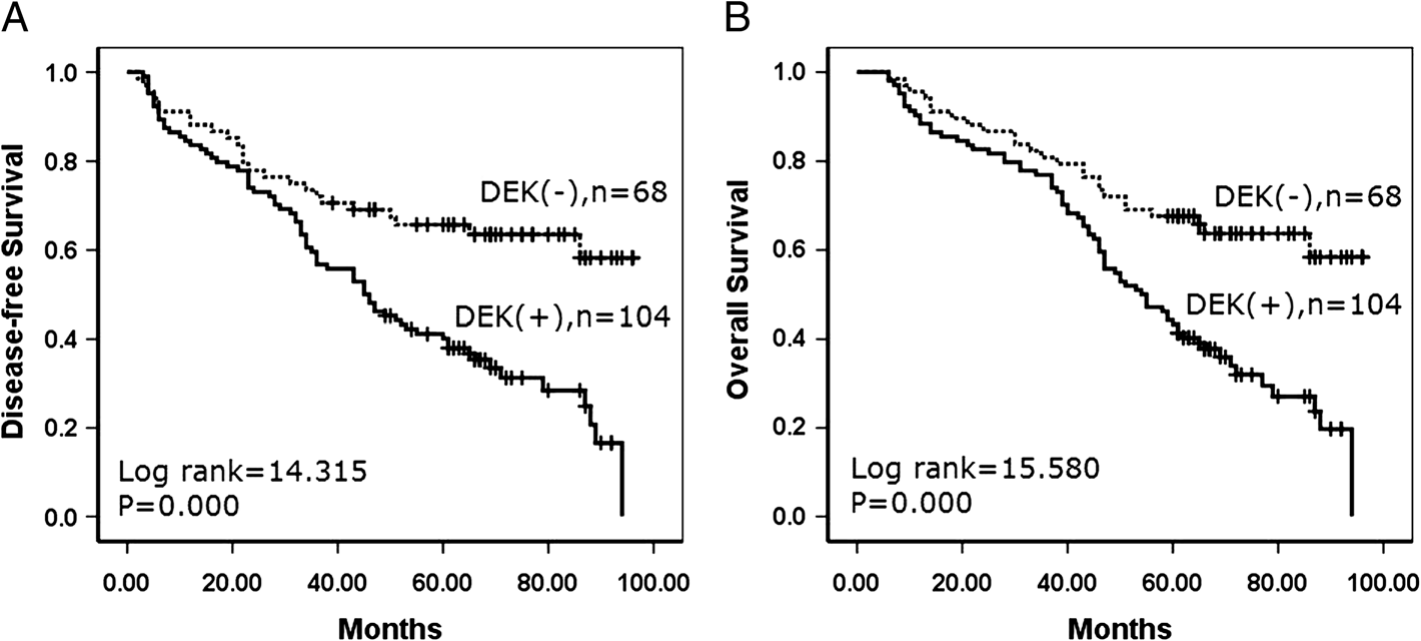
**Kaplan–Meier analysis of disease-free and overall survival rate in 172 gastric cancer patients in relation to DEK protein expression.** Gastric cancer patients with DEK-positive expression had lower disease-free **(A)** and overall survival **(B)** rates than those with DEK-negative expression as determined using the Kaplan–Meier method. (+, positive; −, negative).

**Figure 4:**
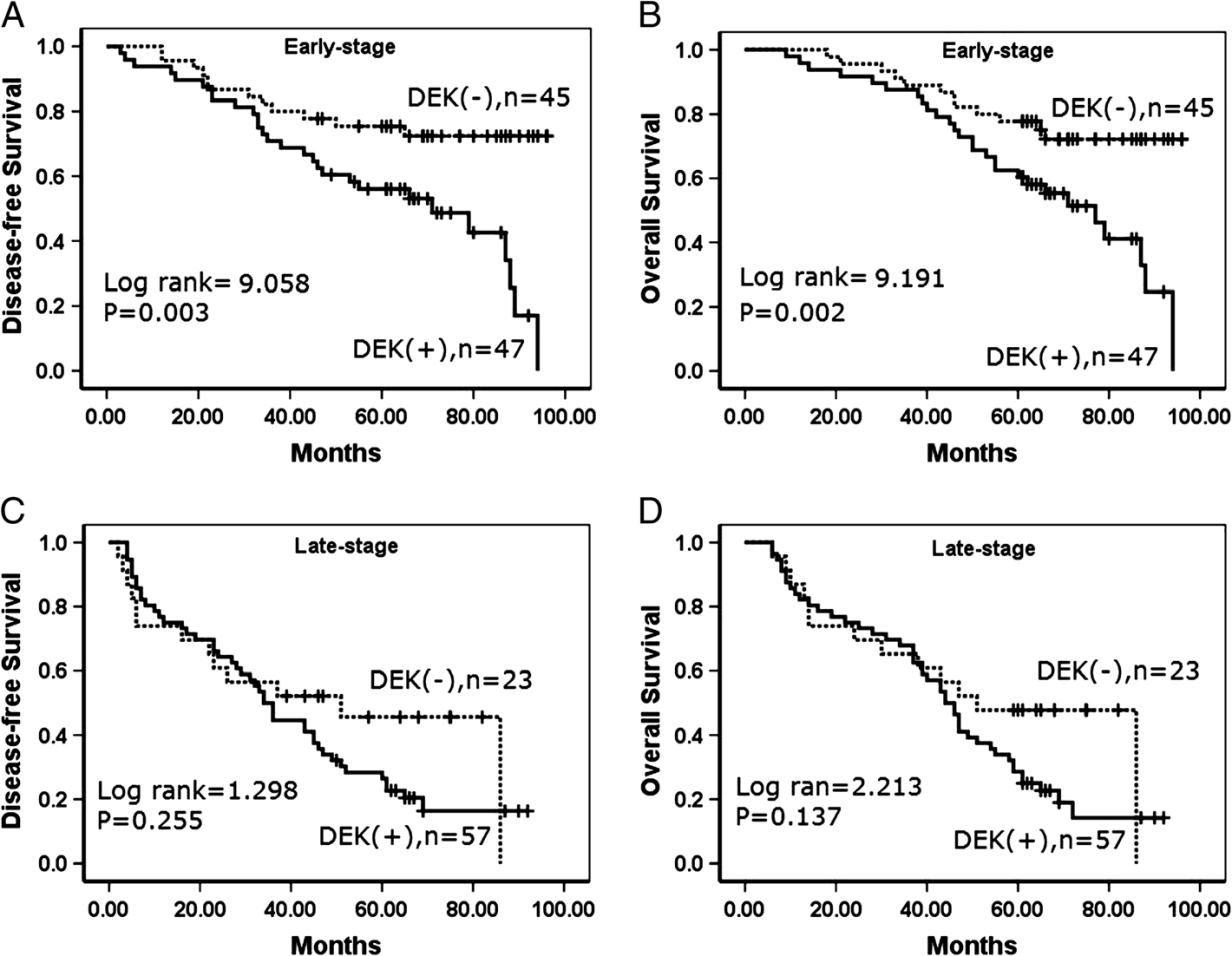
**Kaplan–Meier analysis of disease-free survival and overall survival rates in 172 patients with or without DEK expressed gastric cancer in relation to clinical stage. A–B**: In the early-stage, DEK expression negatively correlated to disease-free survival and overall survival rates, respectively. **C-D**: In the late-stage, the survival rates did not correlated to DEK expression.

A total 172 patients with gastric cancer were identified for analysis of prognostic evaluation. Both disease-free survival and overall survival rates were significantly higher in gastric cancer patients with low DEK expression than in those with high DEK expression (Figure 3). Of the 172 gastric cancer patients, 92 were early stage and 80 were advanced stage. For patients with early stage (I–II) gastric cancer, the survival analysis demonstrated that a DEK level was associated with lower disease-free and overall survival rates (*P* = 0.003 and *P* = 0.002, respectively, log-rank) (Figure 4). However, the expression status of DEK protein did not correlate to survival rate in patients with advanced stage (III–IV) gastric cancer (*P* = 0.255 and *P* = 0.137, respectively, log-rank).

### DEK overexpression is an independent prognostic factor in gastric cancers by Cox proportional hazard regression model

Using univariate analysis, we found that gastric cancer patients with high DEK expression had significantly lower disease-free and overall survival rates than those with low DEK expression tumors. Additionally, serosal invasion, lymph node metastasis, and tumor stage were also associated with disease-free and overall survival rates, suggesting that DEK could be a valuable prognostic factor in gastric cancer. Therefore, multivariate analysis was performed using the Cox proportional hazards model for all significant variables examined in the univariate analysis. We found that the presence of lymph node metastasis (hazard ratio (HR): 1.490, 95% confidence interval (CI): 0.091–2.036, *P* = 0.012) and late stage (HR: 1.837, 95% CI: 1.323–2.551, *P* = 0.000) proved to be independent poor prognostic factors for survival in gastric cancer. Importantly, high DEK expression also emerged as a significant independent poor prognostic factor in gastric cancer (HR: 1.422, 95% CI: 1.033–1.956, *P* = 0.031) (Table 3).

**Table 3:** Cox regression model analysis of various factors in 172 patients with gastric cancer

**Characteristics**	**B**	**SE**	**Wald**	**HR**	**95% CI**		***P *** **value**
					**Lower**	**Upper**	
**Univariate**							
Age	0.308	0.158	3.808	1.360	0.999	1.853	0.051
Gender	0.135	0.155	0.750	1.144	0.844	1.551	0.386
Tumor size	0.306	0.159	3.688	1.358	0.994	1.856	0.055
Serosal invasion	0.397	0.157	6.343	1.487	1.092	2.024	0.012*
Lauren types	0.135	0.118	1.318	1.145	0.909	1.442	0.251
Differentiation	0.219	0.116	3.584	1.245	0.992	1.561	0.058
LN metastasis	0.556	0.156	12.712	1.744	1.285	2.368	0.000**
Clinical stage	0.810	0.160	25.619	2.247	1.642	3.074	0.000**
DEK	0.455	0.160	8.111	1.576	1.152	2.155	0.004**
**Multivariate**							
Serosal invasion	0.224	0.162	1.904	1.251	0.910	1.718	0.168
Clinical stage	0.608	0.167	13.187	1.837	1.323	2.551	0.000**
LN metastasis	0.399	0.159	6.272	1.490	1.091	2.036	0.012*
DEK	0.352	0.163	4.671	1.422	1.033	1.956	0.031*

B: coefficient; SE: standard error; Wald: Wald statistic; HR: hazard ratio;

CI: confidence interval

*P < 0.05, **P < 0.01.

## Discussion

The human DEK gene is generally considered a proto-oncogene because of its involvement in chromosomal translocation in AML and its upregulation in a variety of human malignancies [6,18]. To date, accumulating evidence from studies suggests a correlation between DEK and several types of human malignancies, such as melanoma [19], glioblastoma [20], breast cancer [21] and bladder cancer [22], but little is known about gastric cancers. This is the first study, to our knowledge, to correlate DEK levels in GAC with histological prognostic factors to understand the role of DEK upregulation in gastric cancer progression. Here we performed IF and IHC staining of DEK protein and survival data analysis using 36 of gastric dysplasia and 172 of GAC and their adjacent normal tissue counterparts. We found that high levels of DEK expression were associated with poor prognosis in gastric cancer patients. We also observed that altered expression levels of DEK protein in gastric cancer tissues, which were significantly higher than both adjacent noncancerous tissues and normal stomach tissues. Our results suggest the important role of DEK protein in the prognosis of patients with gastric cancer.

As DEK may be present at higher levels in immature cells than in differentiated counterparts [11], it could also aid in gauging the differentiation potential of tumor cells. Our previous data [15] showed that DEK protein was strongly positive in colorectal cancers and dysplastic adenoma of colon, but negative in adjacent normal mucosa, demonstrating that DEK protein expression levels might be used as a biomarker for early diagnosis of colorectal cancers. Khodadoust *et al.* reported that DEK expression levels can distinguish benign nevi from malignant melanomas, indicating that this protein may prove to be highly useful for differentiating diagnosis [19]. Additionally, Kappes *et al.* investigated the localization of DEK throughout the cell cycle and found it was always on chromatin and as a component of mitotic chromosomes [23]. Here we found that DEK protein is strictly located in the nucleus of gastric cancer cells using IF and IHC staining, and the expression level of DEK is significantly upregulated in gastric cancer and dysplasia than in adjacent normal gastric mucosa, indicating that DEK upregulation is an early event in the progression of gastric cancer.

Despite the strong association between DEK expression and cancer, reports of DEK expression-based outcome in tumor patients are limited. Our previous study [15] reported that high DEK expression is associated with serosal invasion, lymph node metastasis, tumor size and differentiation, which are crucial histological features associated with poor prognosis in colorectal cancer. Also, DEK overexpression concomitant with any of these features correlated with significantly lower 5-year survival rates than those without DEK expression. Consistent with this report, in 2013 Wang *et al*. revealed that acute myeloid leukemia patients with low DEK expression had higher overall survival rates compared with patients with high DEK expression [24]. Similarly, Liu *et al*. reported that DEK protein showed higher expression levels in < 3- year disease-free survival breast cancers patients than it did in ≥3-year disease-free survival patients [21]. Here we also found that DEK expression was strongly associated with survival rates in early-stage tumors, and was significantly higher in DEK low-expressed patients than in DEK high-expressed patients. Thus, IHC examination of DEK could be used as an additional tool to identify gastric cancer patients at risk of malignant progression, and the DEK expression analysis may also be useful in optimizing individual gastric cancer therapy management, favoring a more aggressive regimen in tumors with high DEK expression.

Notably, many studies have reported that DEK implicated in several signaling pathways in tumor cells and played important role in cancer progression. Wise-Draper *et al*. reported that DEK delay differentiation of keratinocyte in a p53-independent way [25]. Wise-Draper *et al*. also reported DEK to act as a negative regulator of p53 activities in a manner that influenced cellular survival [26]. Sandén *et al*. reported that the DEK–NUP214 fusion gene increased the proliferation of myloid cell proliferation through upregulation of mTOR [27]. These data suggested that altered DEK expression patterns might regulate a certain signaling pathway to play an oncogenic role in cancer development and progression. Perhaps, the pathways regulated by DEK may represent a new strategy for cancer therapies and further study is also required to find out the exact signaling pathway regulated by altered DEK in gastric cancer progression.

In conclusion, DEK overexpression appears to be associated with gastric cancer progression, and DEK may potentially be used as a biomarker for prognostic evaluation and as a therapeutic target in gastric cancer.

## Competing interests

The authors declare that they have no competing interests.

## Authors’ contributions

PJ, SY and LS participated in the study conception, design and case selection. LY, CX and PY carried out case collection. PJ, LY, and LZ performed experiments and wrote the manuscript. All the authors read and approved the final manuscript.
